# Carvacrol-induced apoptosis via tumor suppressor gene activation and oxidative stress modulation in a rat model of breast cancer

**DOI:** 10.1007/s12672-025-04149-9

**Published:** 2025-12-10

**Authors:** Amany Elwakkad, Amina A. Gamal el Din, Mohamed A. Hebishy, Howida S. Abou-Seif

**Affiliations:** 1 Medical Physiology Department, Medical Research and Clinical Studies Institute, National Research Centre, Cairo, Egypt; 2 Pathology Department, Medical Research and Clinical Studies Institute, National Research Centre, Cairo, Egypt; 3 Medical Physiology Department, Medical Research and Clinical Studies Institute, National Research Centre, 33 El Buhouth St, Ad Doqi, Dokki, Cairo Governorate, Cairo, Egypt

**Keywords:** Carvacrol, Breast cancer, Apoptosis, Tumor suppressor genes, Oxidative stress

## Abstract

**Objective:**

Breast cancer remains a significant global health challenge, despite advancements in chemotherapy. Targeted therapies utilizing plant-derived compounds are gaining attention, with Carvacrol—a monoterpene phenol—showing promise as an anticancer agent. This study evaluates Carvacrol antioxidant, anti-inflammatory, and pro-apoptotic effects in a DMBA-induced breast cancer rat model.

**Method:**

Female rats were assigned to five groups: a normal/ healthy control (G1), a DMBA-induced cancer group monitored for 120 days (GII), and three tumor-bearing groups receiving Carvacrol (100 mg/kg b.w.) via oral (GIII), intraperitoneal (GIV), or combined routes (GV), administered thrice weekly for 12 weeks.

**Results:**

Carvacrol treatment markedly counteracted the detrimental effects induced by DMBA. Intraperitoneal administration produced the strongest therapeutic response, significantly enhancing apoptotic markers (cytochrome c, TNF-α, FADD) while suppressing anti-apoptotic proteins (Bcl-2, DR2). Pro-apoptotic regulators (p53, p73, TRAIL) were up regulated, confirming activation of the intrinsic apoptotic pathway. Carvacrol also attenuated lipid peroxidation by reducing malondialdehyde (MDA) levels, while boosting total antioxidant capacity and improving inflammatory status. Moreover, restoration of liver and kidney function was observed through normalization of serum ALT, AST, urea, and creatinine levels, together with improved histopathological architecture. Although the intraperitoneal route achieved the greatest efficacy, all routes of administration produced significant improvements compared with the untreated DMBA group, highlighting both the therapeutic potential of Carvacrol and the importance of bioavailability. Overall, Carvacrol suppresses DMBA-induced breast cancer through activation of intrinsic and extrinsic apoptotic pathways, reduction of oxidative stress, and protection of vital organs, with the injection route demonstrating the highest effectiveness.

**Conclusion:**

The study supports Carvacrol potential as a complementary anticancer agent by enhancing apoptosis and reducing oxidative damage. Further research is required to optimize dosage and delivery for clinical applications.

**Graphical abstract:**

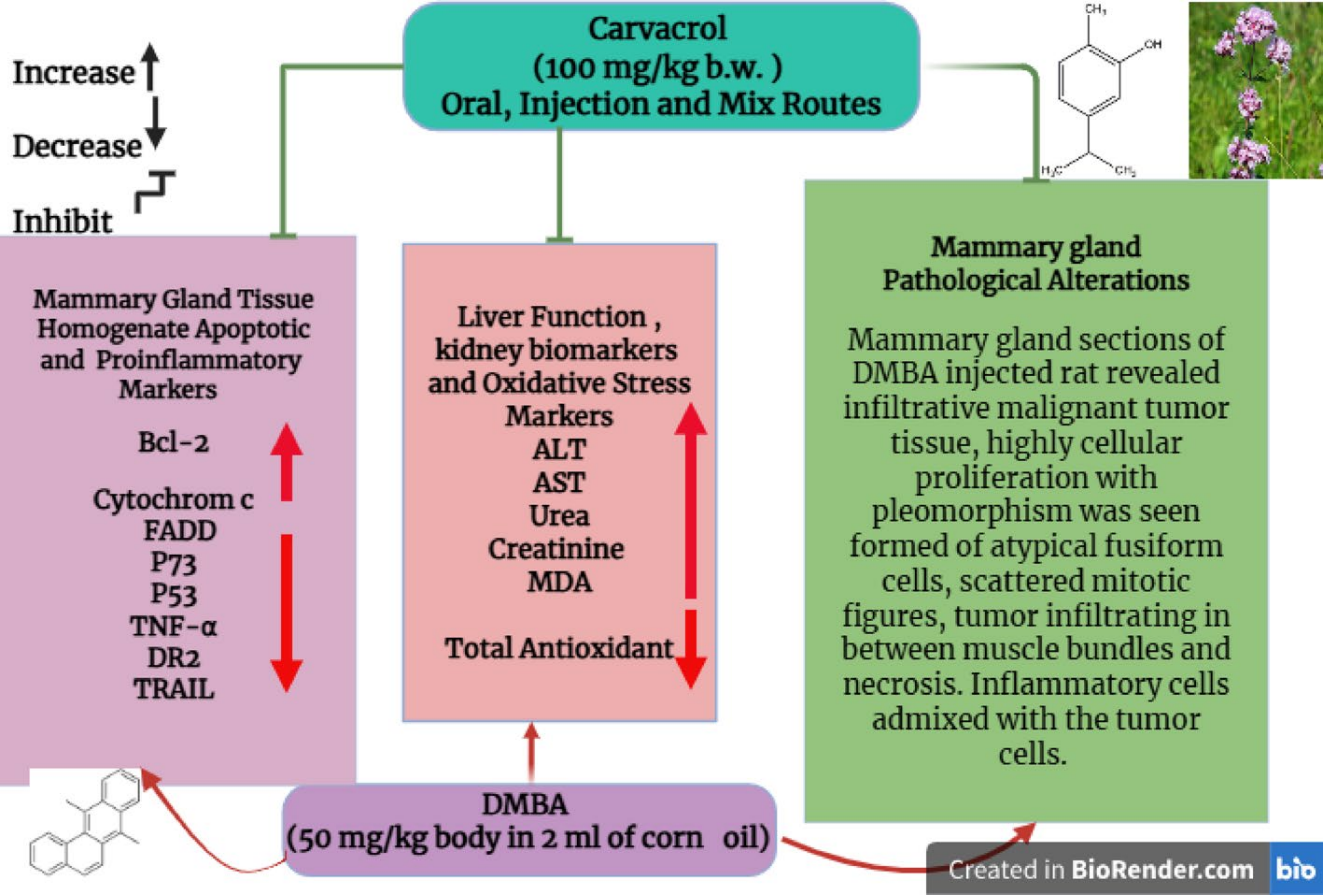

## Introduction

According to the most recent Breast Cancer Statistics report for 2024 [1], approximately 310,720 new cases of invasive breast cancer and 56,500 cases of ductal carcinoma in situ (DCIS) are expected to be diagnosed among U.S. women, with an estimated 42,250 deaths. The majority of invasive breast cancer cases (84%) and deaths (91%) occur in women aged 50 years and older, with about half (52%) of all deaths occurring among those aged 70 years or older [1]. The median age at diagnosis is 62 years overall, but it is lower among Hispanic (57 years), Asian American and Pacific Islander (AAPI; 57 years), Black (60 years), and American Indian/Alaska Native (AIAN; 60 years) women compared with White women (64 years), partly reflecting the younger age distribution in these populations [2]. The median age at death from breast cancer is 69 years overall, ranging from 63 to 64 years among Hispanic, AAPI, and Black women to 70 years among White women [3]. Although breast cancer predominantly affects women, an estimated 2,790 new cases and 530 deaths (approximately 1% of all breast cancer cases and deaths) are expected among men in 2024 [1]. These statistics emphasize the ongoing global and national burden of breast cancer, highlighting the need for effective preventive and therapeutic strategies.

The pathogenesis of breast cancer is influenced by multiple mechanisms, among which oxidative stress plays a pivotal role. Excessive production of reactive oxygen species (ROS), together with impaired antioxidant defenses, contributes to DNA damage, genomic instability, and dysregulated cellular proliferation. These events collectively foster tumor initiation, progression, and metastasis, while reshaping the tumor microenvironment to support malignant growth [4–7]. Experimental models have been essential for understanding these processes, with dimethylbenzanthracene (DMBA) serving as a potent chemical carcinogen that recapitulates key pathogenic events observed in human breast cancer. Upon metabolic activation by cytochrome P450 enzymes (CYP1A1 and CYP1B1), DMBA is converted into reactive intermediates such as DMBA-3,4-diol-1,2-epoxide, which covalently bind to DNA, generating adducts and mutations in oncogenes and tumor suppressor genes [8]. In parallel, DMBA metabolism generates ROS, amplifying oxidative stress, lipid peroxidation, and secondary DNA damage [9]. Together, these genotoxic and oxidative insults activate oncogenic pathways—including c-myc, cyclin D1, and the aryl hydrocarbon receptor (AhR)—promoting cellular proliferation, inhibiting apoptosis, and driving mammary tumorigenesis [10]. By linking epidemiological risk factors to molecular mechanisms, this model provides a robust framework for evaluating potential chemopreventive and therapeutic interventions. Currently available treatments for breast cancer include chemotherapy, endocrine therapy, and targeted biological therapies such as human epidermal growth factor receptor 2 inhibitors [11]. Despite their proven clinical benefits, these therapies are often associated with significant toxicity, high cost, and the development of resistance, which limit their long-term efficacy [12]. These limitations have prompted increasing interest in natural products, particularly phytochemicals, as safer and more accessible complementary or alternative therapeutic strategies. Plant-derived compounds with antioxidant, anti-inflammatory, and pro-apoptotic properties, such as polyphenols, have shown encouraging results in both preclinical and clinical studies [13–18]. Such compounds can modulate multiple pathways involved in cancer initiation and progression, including inhibition of cell proliferation, modulation of estrogen receptor activity, induction of apoptosis, and enhancement of antioxidant defenses [3–6].

Among natural compounds of interest, Carvacrol (2-methyl-5-(1-methylethyl) phenol), a bioactive monoterpenoid phenol predominantly found in the essential oils of oregano (*Origanum vulgare*) and thyme (*Thymus vulgaris*), has garnered attention for its broad biological activities [18]. Carvacrol exhibits antimicrobial, antioxidant, anti-inflammatory, neuroprotective, and anticancer effects [19]– [20], and has been shown to modulate key signaling pathways implicated in tumor development, including angiogenesis, apoptosis, inflammation, and cellular proliferation [21–23]. Its safety as a food additive further supports its potential translational applicability [24]. Preclinical studies have demonstrated the chemopreventive and therapeutic potential of Carvacrol in several malignancies, including breast cancer, melanoma, hepatocellular carcinoma, cervical cancer, and non-small cell lung cancer [25].

Despite growing evidence of Carvacrol diverse biological activities, its therapeutic efficacy and safety in breast cancer remain insufficiently explored. This knowledge gap highlights the need for further preclinical validation using relevant animal models. Therefore, the present study was designed to investigate the anticancer potential of Carvacrol in a DMBA-induced breast cancer rat model. Specifically, the study aimed to: (1) evaluate the therapeutic efficacy of Carvacrol against breast cancer development, (2) explore its underlying biological mechanisms with a focus on oxidative stress, inflammation, and apoptosis, (3) assess its immunomodulatory and antioxidant effects, and (4) examine its safety profile by analyzing liver and kidney function markers. By linking epidemiological insights, mechanistic pathways, and the rationale for using Carvacrol, this introduction now presents a cohesive narrative that flows logically from disease burden to experimental strategy.

## Methods and materials

### Experimental animals

The study employed female Sprague-Dawley albino rats weighing 120–130 g at the start of the experiment. The animals were obtained from the Animal House of the National Research Centre, Dokki, Giza, Egypt. All rats underwent a one-week acclimatization period in plastic cages under standard 12-hour light/dark cycles to adapt to the laboratory environment before experimental procedures commenced. During the entire study, the animals had free access to purified water and a standard commercial diet. Environmental conditions, including temperature, humidity, and light exposure, were carefully controlled and maintained consistently throughout the experimental period. All animal procedures were reviewed and approved by the Institutional Animal Ethics Committee of the National Research Centre (Approval No. 19–204) and conducted in accordance with the guidelines of the National Health and Medical Research Council.

### Tumor induction

A one-week acclimatization period concluded before the rats received subcutaneous injections of 7,12-dimethylbenz[α] anthracene (DMBA) purchased from Sigma Chemicals Company, United States. The animals received a single subcutaneous dose of 50 mg/kg body weight of DMBA dissolved in 2 ml of corn oil, injected into the mammary gland region. This dose was selected based on previous studies [4, 26], where it has been shown to reliably induce mammary tumors in rats within 84–120 days, providing a reproducible breast cancer model.

To monitor tumor induction, animals were weighed weekly and palpated to detect the appearance of tumor nodules throughout the 120-day observation period. Only rats that developed palpable tumors were included in the experimental groups, whereas those without tumor development were excluded from subsequent analyses. Histopathological confirmation was performed at the end of the study to validate tumor formation and characterize tumor morphology.

**Fig. 1:**
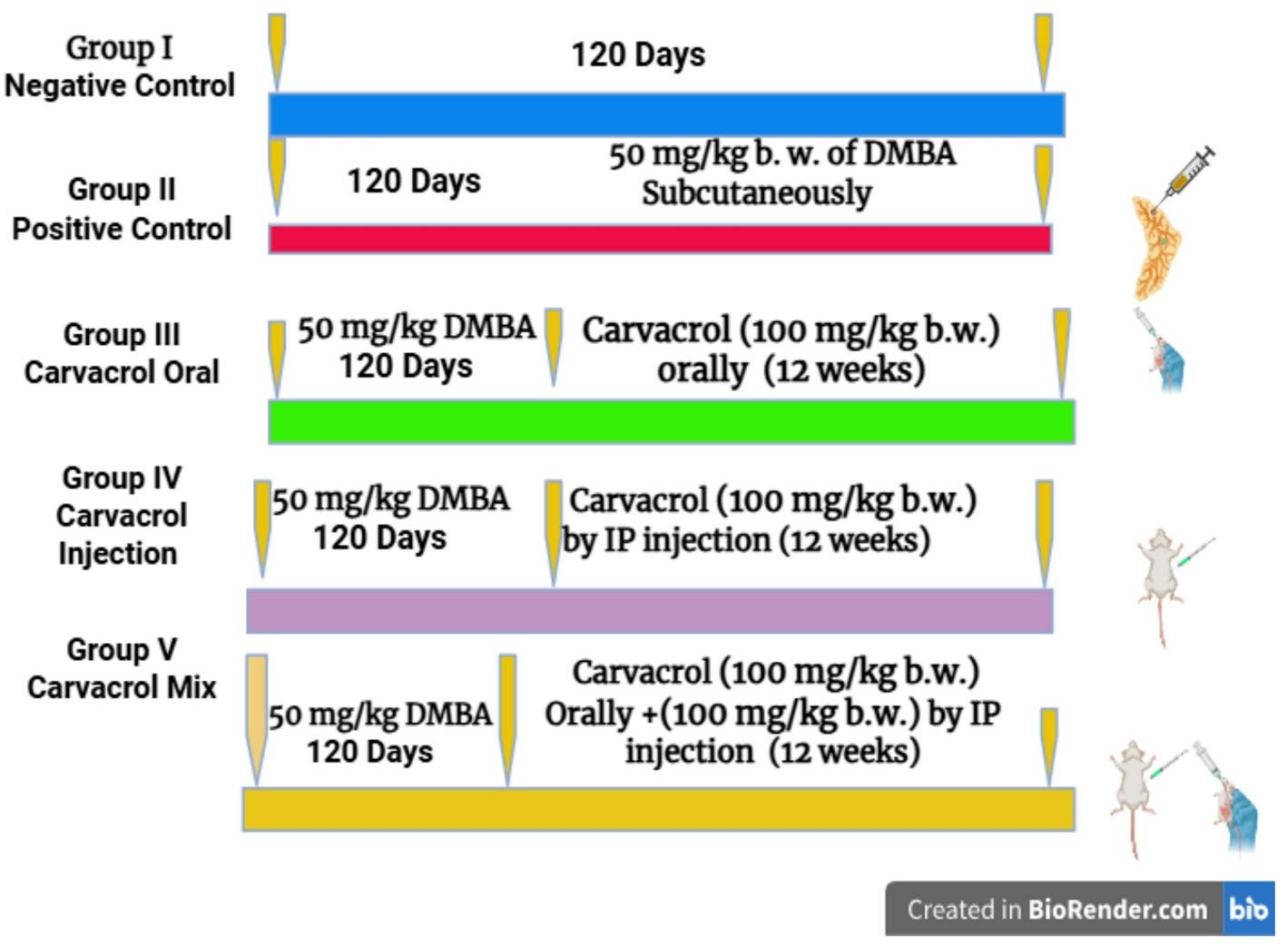
Visual diagram of animal grouping and study design

### Experimental protocol and drug administration

The research used different treatment groups to test how Carvacrol (*Origanum vulgare* ‘oregano’) affects tumor progression in rat subjects (Fig. 1).

I. Group I: Negative control - normal rats without tumors.
II. Group II: The positive control rat in group 2 received 50 mg/kg b. w. of DMBA (in 2 ml of corn oil) administered through subcutaneous injection to the mammary gland. This allowed tumor development for 120 days [4, 26].
III. Group III: Rats with tumors received Carvacrol (100 mg/kg body weight “10% Carvacrol + 75% Tween 80 + 15% ethanol”) orally three times per week for 12 weeks [27].
IV. Group IV: Tumor-bearing rats received Carvacrol (100 mg/kg body weight “10% Carvacrol + 75% Tween 80 + 15% ethanol”) by intraperitoneal injection three times per week for 12 weeks [27].
V. Group V: Tumor-bearing rats received Carvacrol using both routes (oral and intraperitoneal, each equivalent to 100 mg/kg b.w.) three times per week for 12 weeks [27]. A one-hour interval was maintained between the two administration routes to ensure pharmacological distinction.

#### Sampling

After 120 days, rats in groups 1 and 2 were sacrificed, while those in groups 3, 4, and 5 were sacrificed after 12 weeks post-treatment. Blood samples were obtained from the retro-orbital plexus of rats while they were anesthetized with pentobarbital, using sterile tubes. Blood samples were allowed to coagulate before being centrifuged at 3000 rpm for 10 min using a Hettich centrifuge (Newtown, Connecticut, USA). The resulting sera were separated and stored at − 80 °C for biochemical assay analyses.

### Tissue homogenates setup

Tissue homogenates were prepared as follows: normal, treated and untreated tumor-bearing rats, were sacrificed. Breast tissue samples (5 μm) were excised, weighed and rinsed with ice-cold phosphate-buffered saline (0.01 M, pH 7.4). The tissues were then minced and homogenized in phosphate-buffered saline (9 ml per gram tissue) using a SONICS homogenizer (France, Taguig City 1634, Metro Manila, Philippines) with a glass homogenizer on ice. The resulting homogenates were centrifuged at 3000 rpm for 10 min. Supernatants were collected and stored at − 80 °C for further analyses.

### Biochemical analyses

#### Assessment of liver and kidney, oxidative stress and antioxidant biomarkers

Spectrophotometrically (MY 1345003 spectrophotometer, China), using kits (Table 1) purchased from Reactivos GPL (Barcelona, Espana), serum alanine and aspartate aminotransferases (ALT and AST) activities were estimated as described by Reitman and Frankel’s method [28]; urea and creatinine levels were estimated according to the methods of Patton and Crouch [29] and Bowers and Wong [30], respectively. The Total Antioxidants assay kit from Elabscience (Willich, Germany) tested the solution according to Smith et al. [31] while LPO/MDA measurements followed the procedure described by Ohkawa et al. [32].

**Table 1:** Specifications of liver, kidney, and oxidative stress biomarkers

Biomarker	Unit	Sample type	Dilution	Incubation temperature	Incubation time	Catalog number
ALT (GPT)	U/L	Serum	Not specified	25 °C / 30 °C / 37 °C	3 min (after 1 min pre-incubation)	EZ016LQ / EZ017LQ
AST (GOT)	U/L	Serum	Not specified	25 °C / 30 °C / 37 °C	3 min (after 1 min pre-incubation)	EZ012LQ / EZ013LQ
Urea	mg/dL	Serum	1:50 (urine)	37 °C / 15–25 °C	60 s pre-incubation, read 30–90 s	SU036 / SU037
Creatinine	mg/dL	Serum	1:50 (urine), 1:2 if above linearity	37 °C / 15–25 °C	30–90 s	SU015
MDA	nmol/mL	Homogenate	Not specified	Boiling water bath (95 °C)	30 min	MD 25 29
Total Antioxidant Capacity (T-AOC)	mmol/L	Supernatant	Not specified	Room temperature (~ 25 °C)	20 min	E-BC-K136-M

#### Apoptotic pathway examination

All ELISA assays (Table 2) were performed in duplicates to ensure accuracy, reliability, and reproducibility of the results. Breast tissue homogenate (normal and tumor with or without treatment) levels of Cys c (cystatin c), FADD (Fas-associated death domain), TNF-α (tumor necrosis factor alpha), Bcl-2 (B-cell leukemia/lymphoma 2), DR2 (death receptor 2 “pg/ml”), P73 and P53 (tumor protein gene), and TRAIL (tumor necrosis factor apoptosis inducing ligand) were measured using ELISA technique (UV-2401; Shimadzu, Japan) and rats ELISA reagent kits purchased from SinoGeneClon Biotech Co. Ltd, China.

**Table 2:** Details of apoptotic and inflammatory biomarkers used in the study

Marker	Unit	Concentrations of standard	Catalog No.
Cys c	ng/ml	1.5, 3, 6, 12, 18, 27	SG- 20,555
FADD	ng/ml	0.75, 1.5, 3, 6, 9, 13.5	SG- 21,741
TNF-α	ng/ml	15, 30, 60, 120, 180	SG- 20,350
Bcl-2	ng/ml	15, 30, 60,120,180	SG- 20,401
DR2	pg/ml	75, 150, 300, 600, 900,1350	SG- 21,747
P73	µg/L	125, 250, 500, 1000, 1500, 2250	SG- 21,729
P53	µg/L	60, 120, 240, 480, 720, 1080	SG- 21,729
TRAIL	pg/ml	75, 150, 300, 600, 900, 1350	SG- 21,099

###  Histopathological Preparation

At the end of the experiment, animals were sacrificed. Mammary gland tissue, liver, and kidneys were dissected and extracted from the sacrificed animals. Organ tissues were fixed in 10% buffered formalin for 48 h and were processed through ascending grades of alcohol. Xylene was used for clearing, and then each specimen was embedded in molten paraffin wax at 60 degrees centigrade to prepare the paraffin blocks. All histopathological evaluations were performed in duplicate to ensure accuracy, reliability, and reproducibility of the results. For histopathological study [33], serial Sect. 5 microns thick each were prepared from each block using a Leica RM2245 microtome and stained with hematoxylin and eosin (H&E). Paraffin embedded tissue sections were placed on glass slides and then placed in xylene for 5 min (two changes), followed by transferring the slides to absolute ethanol for 2 min (two changes) then sequential rehydration of the slides by placing them in 95% ethanol for 2 min and then rinsing in distilled water for 2 min.

#### Hematoxylin staining

The slides were submerged in Harris’ hematoxylin solution for 5–10 min and then rinsed in tap water for 1–2 min, followed by differentiation in acid alcohol for 1–2 s and then rinsing quickly in tap water, followed by blueing the slides by dipping them in ammonia water for 30 s and then rinsing the slides in tap water for 5 min.

####  Eosin staining

The slides were submerged in eosin Y solution for 1–2 min, followed by quickly rinsing in distilled water.

#### Dehydration and clearing

Slides were dehydrated by sequential immersion in 70% ethanol, 95% ethanol, and absolute ethanol (2 min each), followed by clearing in xylene for 5 min (two changes).

#### Mounting

A drop of mounting medium (Canada Balsam) was applied to the tissue section and covered with a coverslip, and then the slides were allowed to dry in an incubator.

####  Quality control

A control slide was included with each batch of staining.

#### Microscopic examination

Tissues were examined using an Olympus CX41 research microscope. Each section was examined by two expert pathologists. Blinding the sample identity was applied to avoid any bias. Evaluation of concurrent control animals helps to address differences and to control for interstudy variability in prevalence of background or spontaneous histopathology findings. Randomness was considered during examination and taking photos. The ordinal scoring system was applied to reduce intra- and inter-observer variability.

####  Interpretation

Hematoxylin stains nuclei a blue-purple color, and eosin stains the cell cytoplasm and connective tissue pinkish.

#### The scoring criteria and semiquantitative

#####  Evaluation applied in this study for histopathologic grading

Data measurements in this study used the ordinal scoring system where samples were assigned to a category showing an ordered progression in severity based on the estimated percentage of tissue affected by a lesion, where 0 = no change, 1 = < 25%, 2 = 26–50%, 3 = 51–75% 4 = 76–100% [34].

#### Slide tissue microphotography

CCD digital camera (Olympus SC100) attached to the microscope was used for obtaining photomicrographic sections at several magnifications.

### Statistical analysis

Each experimental group consisted of eight animals (*n* = 8). Data are presented as mean ± standard error of the mean (SEM). Data normality was verified using the Shapiro–Wilk test, and homogeneity of variance was assessed with Levene’s test. One-way analysis of variance (ANOVA) was applied to determine statistical differences among groups, followed by a Paired Samples test and Tukey’s post-hoc test for multiple comparisons. A p- value ≤ 0.05 was considered statistically significant.

## Results

### Biochemical assessment of the apoptotic regulators

#### Cytochrome c (pg/ml)

**Fig. 2:**
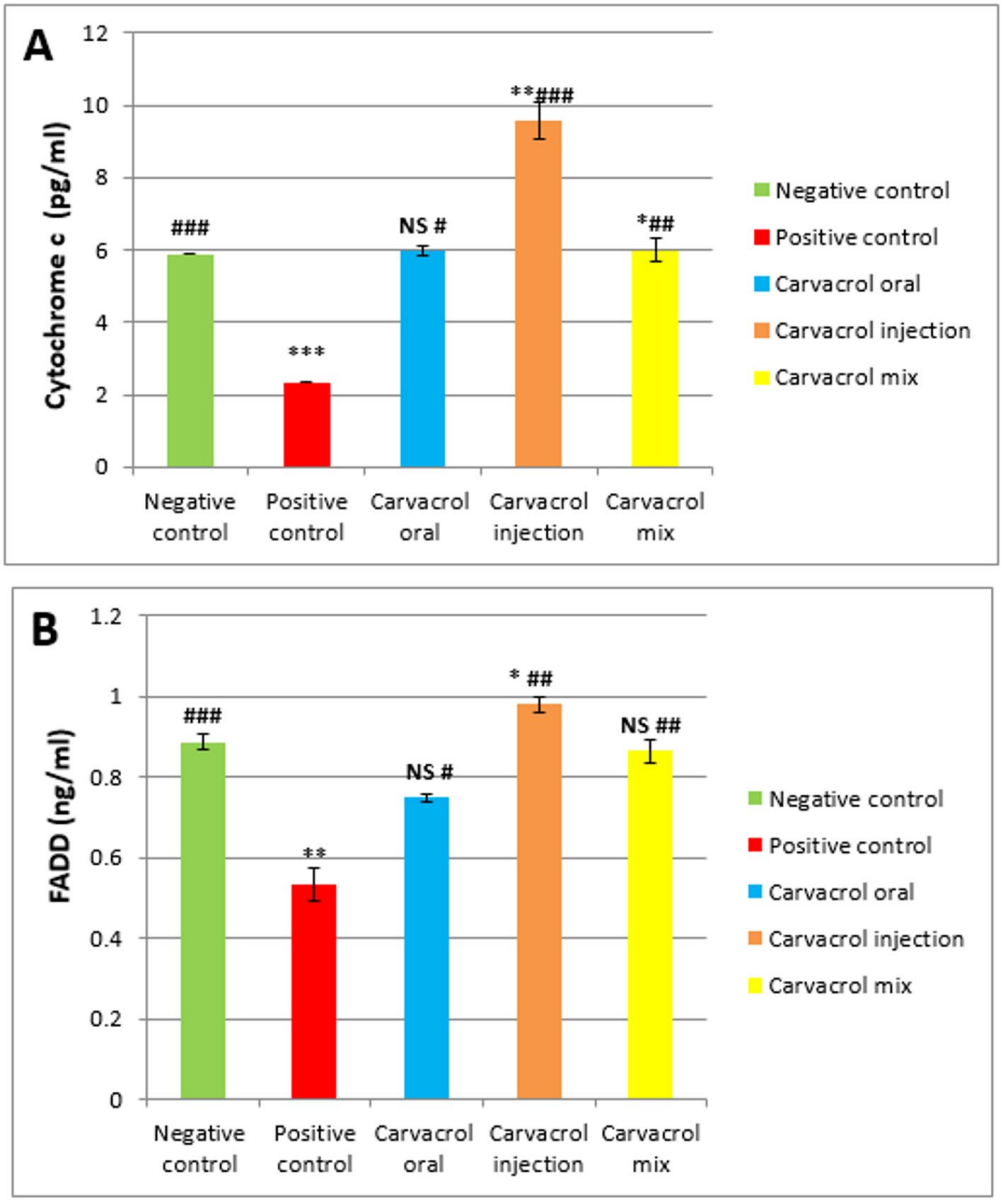
Expressions of cytochrome c and FADD in normal and DMBA- induced breast carcinogenesis and the impact of Carvacrol treatment. Data are presented as mean ± SEM (*n* = 8). *p ≤ 0.05, **p ≤ 0.01, ***p ≤ 0.001 vs. Normal #*p* ≤ 0.05, ##*p* ≤ 0.01, ###*p* ≤ 0.001 vs. Tumor NS: not significant (*p* ≥ 0.05)

Figure 2A demonstrate that untreated breast cancer rats showed significantly reduced cytochrome c levels in their homogenates compared to normal rats (***P ≤ 0.001 vs. normal). Treatment with Carvacrol oral (#*P* ≤ 0.05 vs. tumor and NS vs. normal), injected (### *P* ≤ 0.001 vs. tumor and **P ≤ 0.01 vs. normal), and combined (## *P* ≤ 0.01 vs. tumor and *P ≤ 0.05 vs. normal) markedly enhanced cytochrome c expression. Notably, among the different administration methods, Carvacrol-injected animals resulted in a fourfold increase in cytochrome c levels, exceeding the levels observed in rats treated through oral and combined methods.

##### FADD (ng/ml)

An evaluation of Fig. 2B shows that FADD expression was significantly downregulated in untreated breast cancer rats compared to normal (** *P* ≤ 0.01 vs. normal). Carvacrol-injected therapy markedly elevated FADD expression (## *P* ≤ 0.01 vs. tumor and * *P* ≤ 0.05 vs. normal). Animals treated orally (# *P* ≤ 0.05 vs. tumor and NS vs. normal) or through the combined method (##, *P* ≤ 0.01 vs. tumor; NS vs. normal) also showed elevated FADD expression relative to the tumor group; however, these increases were not sufficient to restore levels to those of the normal control.

###  Tumor suppressor gene expression

#### P73 (µg/L)

**Fig. 3:**
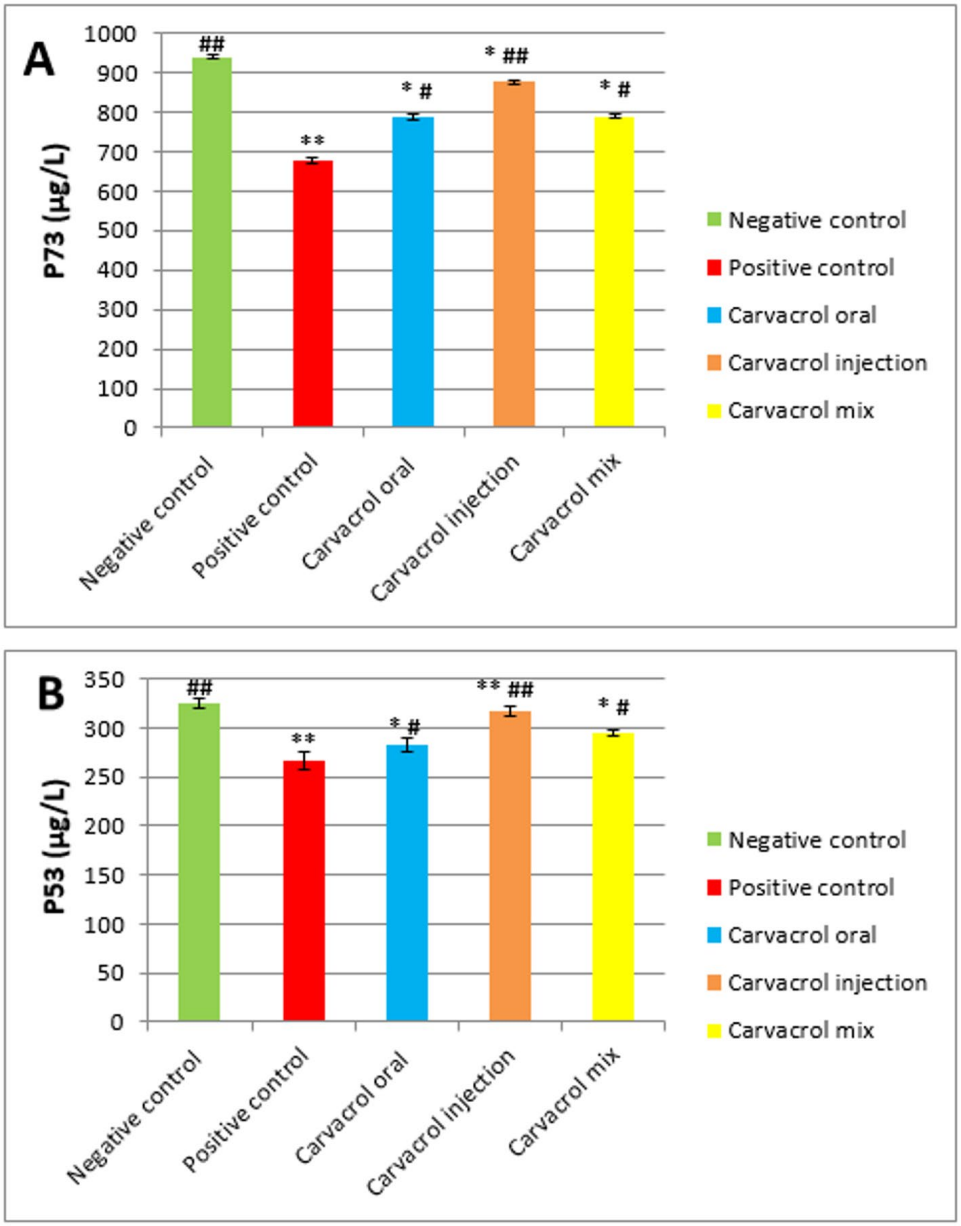
Expressions of P73 and P53 (A& B) in normal and DMBA- induced breast carcinogenesis and the impact of Carvacrol treatment. Data are presented as mean ± SEM (*n* = 8). *p ≤ 0.05, **p ≤ 0.01, ***p ≤ 0.001 vs. Normal #*p* ≤ 0.05, ##*p* ≤ 0.01, ###*p* ≤ 0.001 vs. Tumor NS: not significant (*p* ≥ 0.05)

Research reveals that tumor-bearing rats without treatment demonstrate lower apoptotic P73 levels (**P ≤ 0.01 vs. normal) than healthy rats do but Carvacrol therapy for tumor-bearing rats significantly boosts P73 expression. Rats that received Carvacrol therapy for breast cancer experienced a remarkable rise in P73 expression levels when assessed through injection (## *P* ≤ 0.01 vs. tumor and *P ≤ 0.05 vs. normal), mix and oral (#*P* ≤ 0.05 vs. tumor and *P ≤ 0.05 vs. normal) treatment pathways respectively (Fig. 3A).

####  P53 (µg/L)

The current research revealed downregulated P53 levels (**P ≤ 0.01 vs. normal) in tumor homogenates from untreated rats than from control rats. Carvacrol receiving rat’s up-regulated P53 concentrations markedly that reached their peak in the injected (## *P* ≤ 0.01 vs. tumor and **P ≤ 0.01 vs. normal) as well as oral and mixed groups (#*P* ≤ 0.05 vs. tumor and *P ≤ 0.05 vs. normal) when compared to tumor-bearing rats (Fig. 3B).

### Apoptosis regulator

#### TNF-α (ng/ml)

**Fig. 4:**
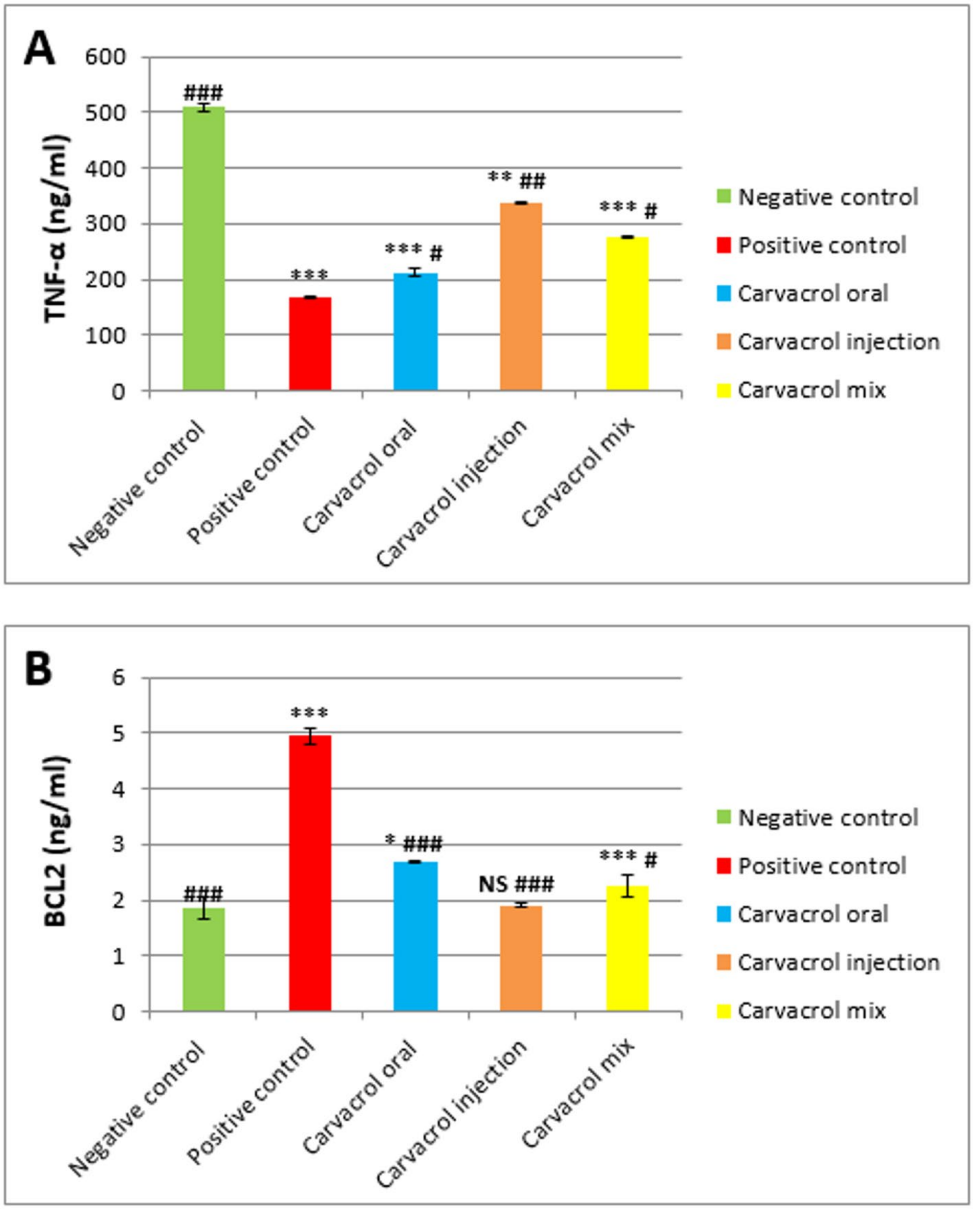
Expression of TNF-α and BCL2 in normal and DMBA- induced breast carcinogenesis and the impact of Carvacrol treatment. Data are presented as mean ± SEM (*n* = 8). *p ≤ 0.05, **p ≤ 0.01, ***p ≤ 0.001 vs. Normal #*p* ≤ 0.05, ##*p* ≤ 0.01, ###*p* ≤ 0.001 vs. Tumor NS: not significant (*p* ≥ 0.05)

Currently, Fig. 4A demonstrates that homogenate TNF-α concentration of tumor-bearing rats is decreased markedly (***P ≤ 0.001 vs. normal) in contrast to normal. Following Carvacrol therapy, NF-α expression significantly increased relative to the untreated tumor bearing animals. The injection route (## *P* ≤ 0.01 vs. tumor and **P ≤ 0.01 vs. normal) of Carvacrol was found to be the most effective compared to oral and mixed groups (#*P* ≤ 0.01 vs. tumor and ***P ≤ 0.001 vs. normal).

#### BCL2 (ng/ml)

Bcl-2 expression was downregulated remarkably in Carvacrol receiving animals relative to those with the untreated breast cancer (***P ≤ 0.001 vs. normal) where Bcl-2 was notably up-regulated. Among different tissue homogenate groups, the treatment with Carvacrol through injection (###*P* ≤ 0.001 vs. tumor and NS vs. normal) showed the strongest effect in lowering Bcl-2 protein levels (Fig. 4B) when compared to both oral and combined treatments (##*P* ≤ 0.01 vs. tumor and * *P* ≤ 0.05 vs. normal).

#### DR2 (Pg/mL)

**Fig. 5:**
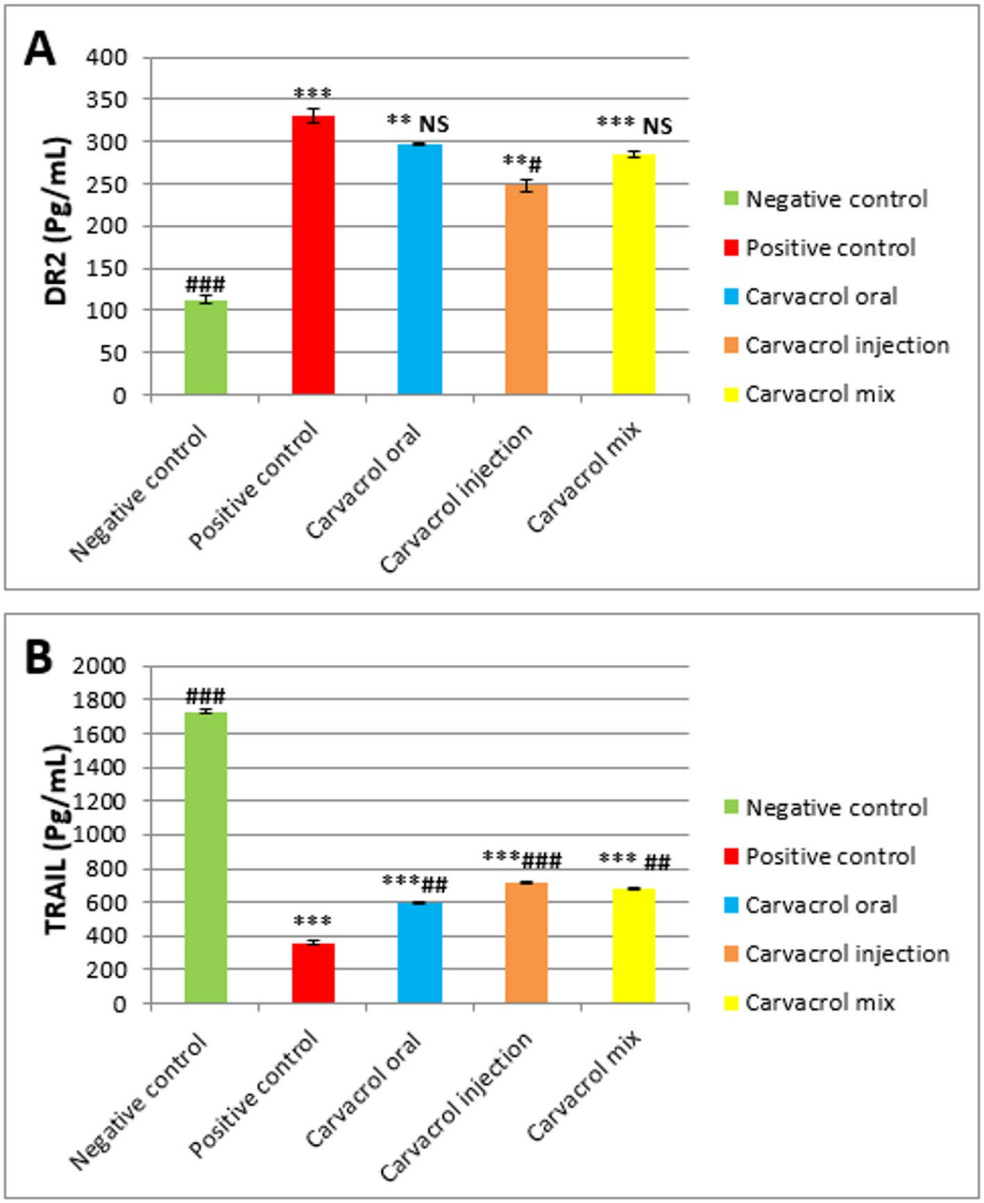
Expression of DR2& TRAIL (A& B) in normal and DMBA- induced breast carcinogenesis and the impact of Carvacrol treatment. Data are presented as mean ± SEM (*n* = 8). *p ≤ 0.05, **p ≤ 0.01, ***p ≤ 0.001 vs. Normal #*p* ≤ 0.05, ##*p* ≤ 0.01, ###*p* ≤ 0.001 vs. Tumor NS: not significant (*p* ≥ 0.05)

The tested rats who received Carvacrol downregulated DR2 expression remarkably (*P* ≤ 0.05) compared the untreated breast cancer rats which elevated DR2 remarkably (***P ≤ 0.001 vs. normal). Results showed that Carvacrol injection (# *P* ≤ 0.05 vs. tumor and **P ≤ 0.01 vs. normal) caused the strongest decrease in DR2 levels among oral (NS vs. tumor and **P ≤ 0.01 vs. normal) and mixed (NS vs. tumor and ***P ≤ 0.001 vs. normal) administration groups in tumor tissue homogenate samples (Fig. 5A).

#### TRAIL (Pg/mL)

Figure 5B indicates that the untreated breast cancer rats exhibited significantly lower (***P ≤ 0.01 vs. normal) levels of TRAIL in contrast to normal rats. However, treating the animals with Carvacrol injected group (### *P* ≤ 0.001 vs. tumor and ***P ≤ 0.001 vs. normal) as well as oral and mixed groups (## *P* ≤ 0.01 vs. tumor and ***P ≤ 0.001 vs. normal) led to a notable elevated or improved TRAIL expression relative to the breast cancer group.

### Biochemical assessment of serum hepatic markers

Serum ALT activity was significantly increased (***P ≤ 0.001 vs. normal) in DMBA-induced group relative to normal. Administration of Carvacrol significantly reduced ALT activity compared with tumor group (Table 3). The Carvacrol-injected and mixed groups (##*P* ≤ 0.01 vs. tumor, NS vs. normal) demonstrated the greatest amelioration of ALT activities relative to oral treatment (#*P* ≤ 0.05 vs. tumor, *P ≤ 0.05).

Likewise, serum AST activity showed a significant elevation (**P ≤ 0.01 vs. normal) in the DMBA-induced breast cancer group compared with normal (Table 3). Treatment with Carvacrol markedly attenuated this elevation relative to the cancer group. Notably, the Carvacrol-injected and mixed groups exhibited the most pronounced improvement in AST activity (#*P* ≤ 0.05 vs. tumor, NS vs. normal) respectively compared with oral treated group (#*P* ≤ 0.05 vs. tumor, *P ≤ 0.05).

**Table 3:** Serum ALT and AST activities in normal and DMBA- induced breast carcinogenesis and the impact of carvacrol treatment

Parameters Groups	ALT (U/L)	Significance relative to normal	Significance relative to tumor	AST (U/L)	Significance relative to normal	Significance relative to tumor
**G1** Normal/ Negative control	208.2 ± 17.9	–	###	231.9 ± 7.2	–	##
**G2** Positive control	713.7 ± 22.9	***	–	436.0 ± 3.7	**	–
**G3** Carvacrol oral	441.4 ± 14.5	*	#	349.6 ± 15.8	*	#
**G4** Carvacrol injection	231.4 ± 12.2	NS	##	257.4 ± 4.0	NS	##
**G5** Carvacrol mix (oral& injection)	247.1 ± 19.6	NS	##	251.9 ± 18.5	NS	#

Data are presented as mean ± SEM (*n* = 8). *p ≤ 0.05, **p ≤ 0.01, ***p ≤ 0.001 vs. Normal #*p* ≤ 0.05, ##*p* ≤ 0.01, ###*p* ≤ 0.001 vs. Tumor NS: not significant (*p* ≥ 0.05)

### Biochemical assessment of serum renal markers

Serum urea levels were significantly elevated (***P ≤ 0.001 vs. normal) in tumor-bearing animals compared with the normal group (Table 4). Treatment with Carvacrol restored urea levels toward normal values relative to the cancer group. Among the treatment modalities, Carvacrol injection exerted the most pronounced effect (##*P* ≤ 0.01 vs. tumor, **P ≤ 0.01), producing a marked reduction in serum urea concentration with oral (NS vs. tumor, ***P ≤ 0.001) and mixed (#*P* ≤ 0.05 vs. tumor, **P ≤ 0.01) treatments.

Similarly, serum creatinine levels were significantly increased (*P ≤ 0.05 vs. normal) in tumor-bearing rats relative to normal (Table 4). Carvacrol treatment markedly improved creatinine levels compared with the cancer group, with the injected form again showing the greatest efficacy in lowering serum creatinine concentrations (###*P* ≤ 0.001 vs. tumor, *P ≤ 0.05) with oral (#*P* ≤ 0.05 vs. tumor, ***P ≤ 0.001) and mixed (##*P* ≤ 0.01 vs. tumor, **P ≤ 0.01) treatments.

**Table 4:** Serum Urea and creatinine concentrations in normal and DMBA- induced breast carcinogenesis and the impact of carvacrol treatment

Parameters Groups	Urea (mg/dl)	Significance relative to normal	Significance relative to tumor	Creatinine (mg/dl)	Significance relative to normal	Significance relative to tumor
**G1** Normal/ Negative control	53.6 ± 1.0	----	###	0.190 ± 0.03	----------	#
**G2** (tumor) Positive control	118.2 ± 3.9	***	------	0.683 ± 0.11	*	----------
**G3** Carvacrol oral	102.2 ± 4.1	***	NS	0.503 ± 0.1	***	#
**G4** Carvacrol injection	70.7 ± 1.1	**	##	0.248 ± 0.02	*	###
**G5** Carvacrol mix (oral& injection)	95.7 ± 2.5	**	#	0.418 ± 0.05	**	##

Data are presented as mean ± SEM (*n* = 8). *p ≤ 0.05, **p ≤ 0.01, ***p ≤ 0.001 vs. Normal #*p* ≤ 0.05, ##*p* ≤ 0.01, ###*p* ≤ 0.001 vs. Tumor NS: not significant (*p* ≥ 0.05)

### Assessment of oxidative stress

Relative to the normal group, DMBA injection caused a considerable elevation in the MDA level of breast tissue homogenate (***P ≤ 0.001 vs. normal). Co-treatment with Carvacrol significantly reduced MDA levels compared with the tumor-bearing group, with the injection route demonstrating the greatest effectiveness (## *P* ≤ 0.01 vs. tumor, *P ≤ 0.05) over oral or mixed (#*P* ≤ 0.05 vs. tumor, **P ≤ 0.01) administration (Table 5).

In parallel, DMBA administration led to a marked reduction (***P ≤ 0.001 vs. normal) in total antioxidant levels relative to the normal group. Carvacrol treatment markedly enhanced total antioxidant levels compared with the breast cancer group (Table 5), with the injected form again showing superior efficacy (## *P* ≤ 0.01 vs. tumor, *P ≤ 0.05) to oral and mixed routes (#*P* ≤ 0.05 vs. tumor, **P ≤ 0.01).

**Table 5:** Total antioxidant and MDA levels in in normal and DMBA- induced breast carcinogenesis and the impact of carvacrol treatment

Parameters Groups	Total antioxidant (U/mL)	Significance relative to normal	Significance relative to tumor	MDA (nmol/g)	Significance relative to normal	Significance relative to tumor
**G1** Normal/ Negative control	18.07 ± 4.8	-----------	###	5.27 ± 1.0	----	###
**G2** Positive control	6.81 ± 0.8	***	----------	14.93 ± 0.4	***	------
**G3** Carvacrol oral	9.57 ± 0.4	**	#	10.07 ± 0.3	**	#
**G4** Carvacrol injection	13.57 ± 0.2	*	##	8.44 ± 0.1	*	##
**G5** Carvacrol mix (oral& injection)	10.70 ± 0.3	**	#	9.11 ± 0.5	**	#

Data are presented as mean ± SEM (*n* = 8). *p ≤ 0.05, **p ≤ 0.01, ***p ≤ 0.001 vs. Normal #*p* ≤ 0.05, ##*p* ≤ 0.01, ###*p* ≤ 0.001 vs. Tumor NS: not significant (*p* ≥ 0.05)

### Histopathological alterations

####  Mammary gland alterations

**Fig. 6:**
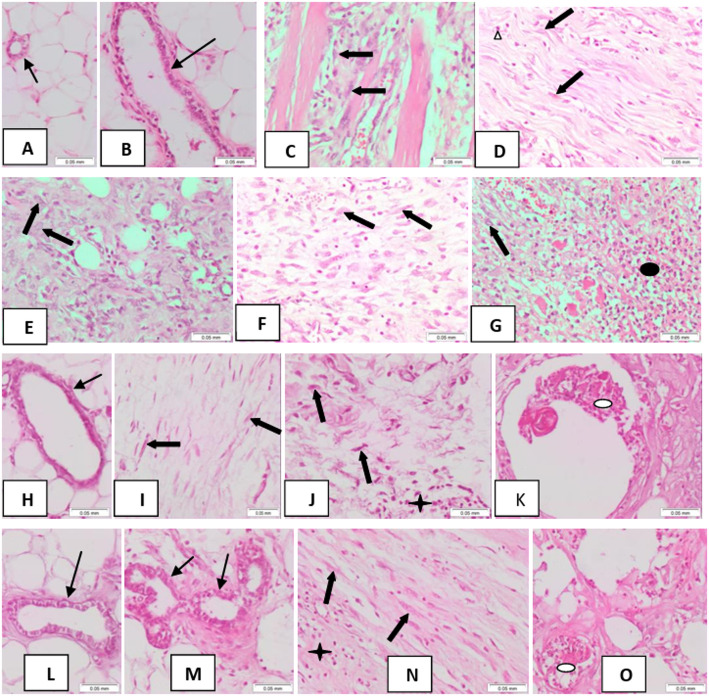
Mammary gland tissue of A&B control female rats showing benign mammary acinus and duct with benign double lining (thin black arrows). C-G DMBA-injected rats showing: C fusiform atypical cell proliferation (score 4) (thick black arrows) infiltrating muscle fibers. D, E, F pleomorphic, tumorous cell proliferation (score 4) (thick black arrows) and mitotic figures (score 4) (white triangle). G necrosis (score 3) (black oval shape) at the right half of the picture with atypical cells seen at the upper left part. H-K DMBA-injected rats treated with oral Carvacrol showing: H, benign mammary duct (thin black arrow); I, J, residual scattered fusiform atypical tumor cells (score 1) (thick black arrows) with inflammatory cells at lower right part (score 2) (black star); K, fat necrosis (score 2) (white oval shape). Figure 6L DMBA-injected rats treated with injected Carvacrol showing benign mammary ducts with benign double epithelial and myoepithelial cell lining (thin black arrow) approximating control. NO tumor (score 0). M-O DMBA-injected rats treated with combined oral and injected Carvacrol showed the following: M benign mammary ducts lined by benign, double lining; N scattered fusiform residual atypical cells (score 1) (thick black arrows) with inflammatory cells at the lower left part (score 1) (black star); O fat necrosis (score 1) (white oval shape). (H&E × 200) **N.B. Scoring system applied. ** N.B. Scoring system applied (0 = no change, 1 = < 25%, 2 = 26–50%, 3 = 51–75%, 4 = 76–100%)

Histopathological examination of mammary gland tissue sections from normal control rats revealed benign mammary acini and ducts (score 0), lined by normal double epithelium and myoepithelium, surrounded by mature adipocytes (Figs. 6A, B). In contrast, mammary gland sections from DMBA-injected rats exhibited extensive infiltrative malignant tumor tissue in all animals (Figs. 6C–G). The lesions were characterized by marked atypia, cellular proliferation, pleomorphism, and frequent mitoses (score 4; Figs. 6D–G), with infiltration between muscle bundles (score 4; Fig. 6C). Tumor necrosis was observed in some areas (score 3; Fig. 6G), accompanied by inflammatory cell infiltration (score 3; Figs. 6D–F) and fat necrosis (score 3).

In rats treated with injected Carvacrol following DMBA administration (Fig. 6L), mammary tissue sections showed no tumor tissue (score 0), absence of atypia (score 0), and no necrosis (score 0), with only minimal inflammatory cell infiltration (score 1) and no fat necrosis (score 0), closely approximating the histology of the normal control group. Oral Carvacrol treatment after DMBA injection (Figs. 6H–K) resulted in mammary tissue sections that displayed largely benign ducts (score 0; Fig. 6H), with residual tumor areas exhibiting minimal pleomorphism and atypia (score 1; Figs. 6I, J). Fat necrosis (score 2; Fig. 6K) and moderate inflammatory cell infiltration (score 2; Fig. 6J) were also observed, while no necrosis was detected within residual tumor tissue (score 0).

In the combined oral and injected Carvacrol treatment group following DMBA injection, mammary tissue sections showed predominantly benign ducts (score 0; Fig. 6M) with residual atypical cells (score 1; Fig. 6N). Fat necrosis was minimal (score 1; Fig. 6O), inflammatory cell infiltration was mild (score 1; Fig. 6N), and no necrosis was observed within the residual atypical cells (score 0). Note: Histopathological scoring system applied as follows: 0 = no change, 1 = < 25%, 2 = 26–50%, 3 = 51–75%, 4 = 76–100%.

#### Liver alterations

**Fig. 7:**
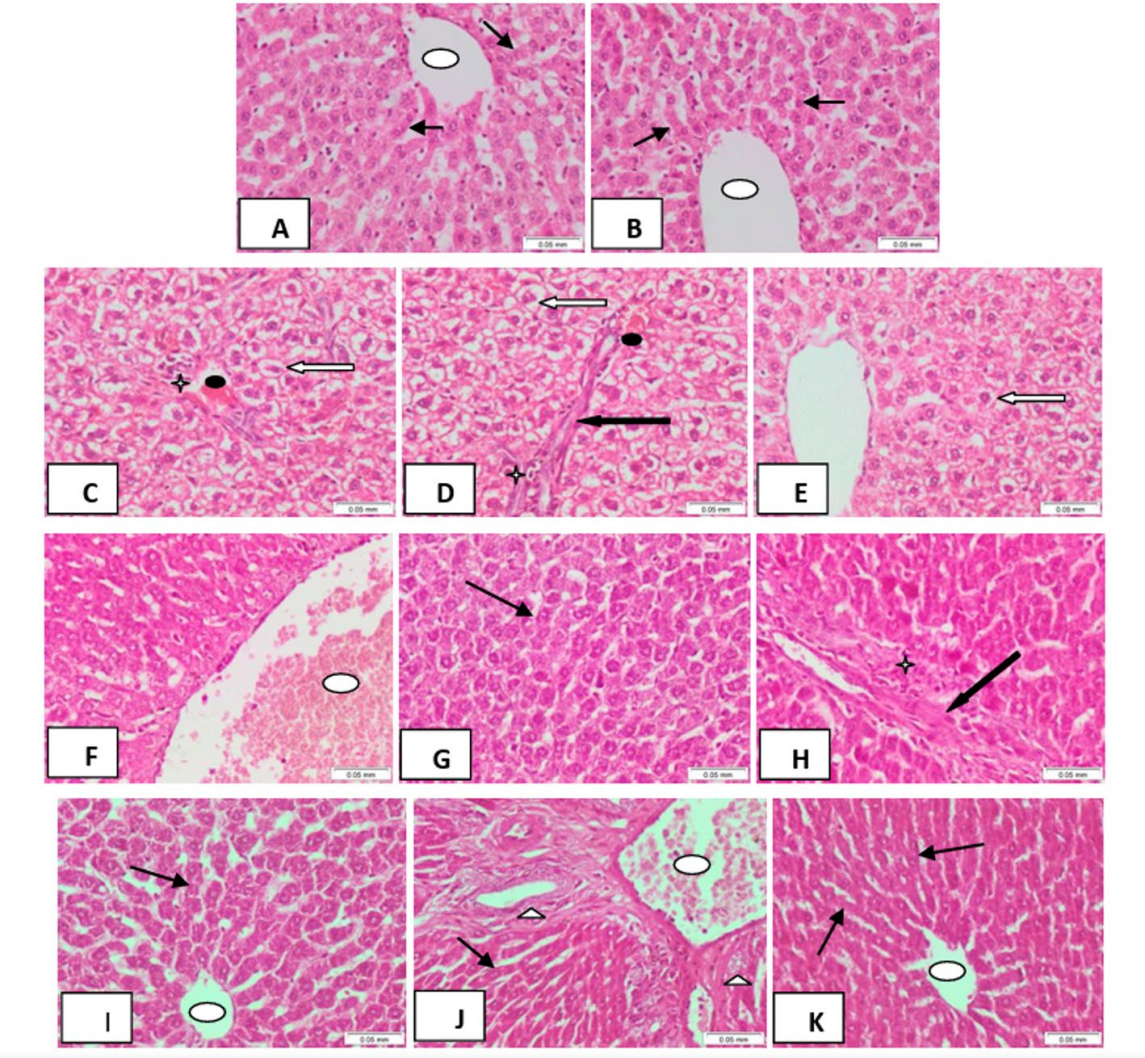
Liver tissue sections of A and B control rats showing ordinary hepatocytes (thin black arrows) arranged in plates of single cell thickness, radiating from the central vein (oval white shape). C-E DMBA-injected rats showed marked hydropic degeneration within liver cells (score 4) (white arrows), sinusoidal congestion (score 2) (black oval shape), inflammatory cell clusters (score 3) (black star), and fibrotic bands within hepatic lobules (score 2) (thick black arrow). F-H DMBA-injected rats treated with oral Carvacrol showed F markedly dilated, congested central vein (white oval shape), G ordinary hepatocytes (thin black arrow), and H residual fibrotic band within hepatic lobule (score 1) (thick black arrow) together with inflammatory cell aggregate (score 2) (black star). I J DMBA-injected rats treated with injected Carvacrol showed I. ordinary hepatocytes (thin black arrow), J. bile duct proliferation (white triangle) (score 1), and dilated central vein (white oval shape). K DMBA-injected rats treated with combined oral and injection Carvacrol showed ordinary hepatocytes (thin black arrows) radiating from the central vein; picture approximating control. (H&E × 200). **N.B. Scoring system applied (0 = no change, 1 = < 25%, 2 = 26–50%, 3 = 51–75%, 4 = 76–100%)

Liver tissue sections from control rats displayed normal hepatic architecture, with polyhedral hepatocytes containing central, rounded basophilic nuclei and abundant eosinophilic cytoplasm. Hepatocyte plates radiated from a central vein, which appeared unremarkable (Figs. 7A, B). In contrast, liver sections from DMBA-injected rats exhibited marked hydropic degeneration within hepatocytes (score 4; Figs. 7C–E), moderate sinusoidal congestion (score 2; Figs. 7C, D), fibrotic bands within hepatic lobules (score 2), and clusters of inflammatory cells (score 3; Figs. 7C, D), indicating DMBA-induced hepatotoxicity.

Oral Carvacrol treatment following DMBA injection resulted in liver sections showing mostly normal hepatocytes without hydropic degeneration (score 0; Fig. 7G). Residual fibrotic bands were minimal (score 1; Fig. 7H), inflammatory cell aggregates were moderate (score 2; Fig. 7H), and the central vein remained markedly dilated and congested (score 3; Fig. 7F), reflecting partial hepatoprotective effects. In the injected Carvacrol group after DMBA exposure, liver sections revealed ordinary hepatocytes without hydropic degeneration (score 0; Fig. 7I), absence of fibrotic bands (score 0; Fig. 7I), minimal bile duct proliferation (score 1; Fig. 7J), and mild dilation of the central vein (score 1; Fig. 7J), approximating normal hepatic architecture.

The combined oral and injected Carvacrol treatment group showed liver tissue sections with preserved hepatocyte morphology, no hydropic degeneration (score 0), absence of fibrotic bands (score 0), no congestion (score 0), no bile duct proliferation (score 0), and no inflammatory cell infiltration (score 0), closely resembling the normal control group (Fig. 7K). Note: Histopathological scoring system applied as follows: 0 = no change, 1 = < 25%, 2 = 26–50%, 3 = 51–75%, 4 = 76–100%.

####  Kidney alterations

**Fig. 8:**
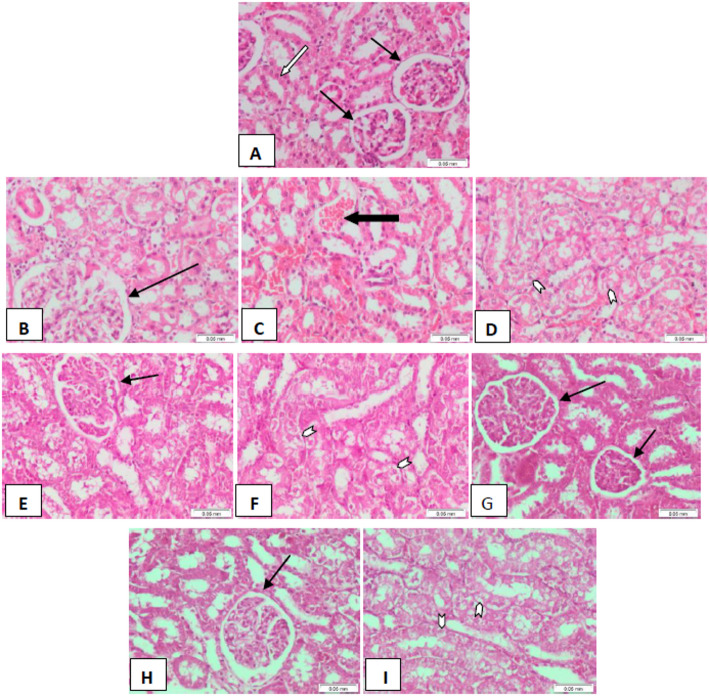
Kidney tissue sections of a control rat showing ordinary, averagely cellular glomeruli (thin black arrows) surrounded by ordinary renal tubules lined by regular epithelium (white arrow). B-D DMBA-injected rats showing: B average cellular glomerulus (thin black arrow), C congestion within renal interstitial tissues (score 3) (thick black arrow), and D vacuolar degeneration within renal tubular epithelium (score 4) (white arrowheads). E, F DMBA-injected rats treated with oral Carvacrol showing E, an average cellular glomerulus (thin black arrow), and F, residual vacuolar degeneration within renal tubules (score 2, white arrowheads). G DMBA-injected rats treated with injected Carvacrol showing average cellular glomeruli (thin black arrows) surrounded by ordinary renal tubules. H, I DMBA-injected rats treated with combined oral and injection Carvacrol showed H average cellular glomerulus (thin black arrow) and I residual vacuolar degeneration within epithelium lining adjacent tubules (score 1) (white arrowheads). (H&E × 200) ** N.B. Scoring system applied (0 = no change, 1 = < 25%, 2 = 26–50%, 3 = 51–75%, 4 = 76–100%)

Kidney tissue sections from control rats showed normally cellular glomeruli, with surrounding renal tubules lined by regular epithelium, indicating preserved renal architecture (Fig. 8A). In contrast, sections from DMBA-injected rats exhibited averagely cellular glomeruli (Fig. 8B) but showed pronounced congestion within the renal interstitial tissue (score 3; Fig. 8C) and severe vacuolar degeneration in the renal tubular epithelium (score 4; Fig. 8D), reflecting DMBA-induced renal toxicity.

In the oral Carvacrol-treated group following DMBA injection, kidney sections displayed averagely cellular glomeruli (Fig. 8E), with no congestion observed in the renal interstitial tissue (score 0). Residual vacuolar degeneration was noted in some renal tubules (score 2; Fig. 8F), indicating partial protective effects of Carvacrol. Similarly, in the injected Carvacrol-treated group after DMBA exposure, kidney sections revealed averagely cellular glomeruli with normally lined renal tubules (Fig. 8G), and neither interstitial congestion nor tubular vacuolar degeneration was observed (score 0), approximating the histology of the control group.

In the combined oral and injected Carvacrol treatment group following DMBA administration, kidney tissue sections showed averagely cellular glomeruli (Fig. 8H) with no congestion in the interstitial tissue (score 0) and minimal residual vacuolar degeneration within renal tubular epithelium (score 1; Fig. 8I). These findings indicate that Carvacrol, particularly in the injected form or in combination, effectively mitigates DMBA-induced renal histopathological alterations.

*Note* Histopathological scoring system applied as follows: 0 = no change,* 1 = < 25%*,* 2 = 26–50%*,* 3 = 51–75%*,* 4 = 76–100%.*

## Discussion

Carvacrol treatment markedly attenuated DMBA-induced breast carcinogenesis, as reflected by improved survival, reduced tumor burden, and modulation of both apoptotic and inflammatory markers. This study comprehensively investigate the impact of Carvacrol on DMBA-induced breast cancer in vivo, highlighting its novelty. Importantly, the injection route produced the most pronounced effects, which may be attributed to enhanced bioavailability and systemic distribution compared with other routes of administration. These findings not only confirm the chemopreventive and therapeutic promise of Carvacrol but also introduce a new line of evidence supporting its potential application in breast cancer management.

**Fig. 9:**
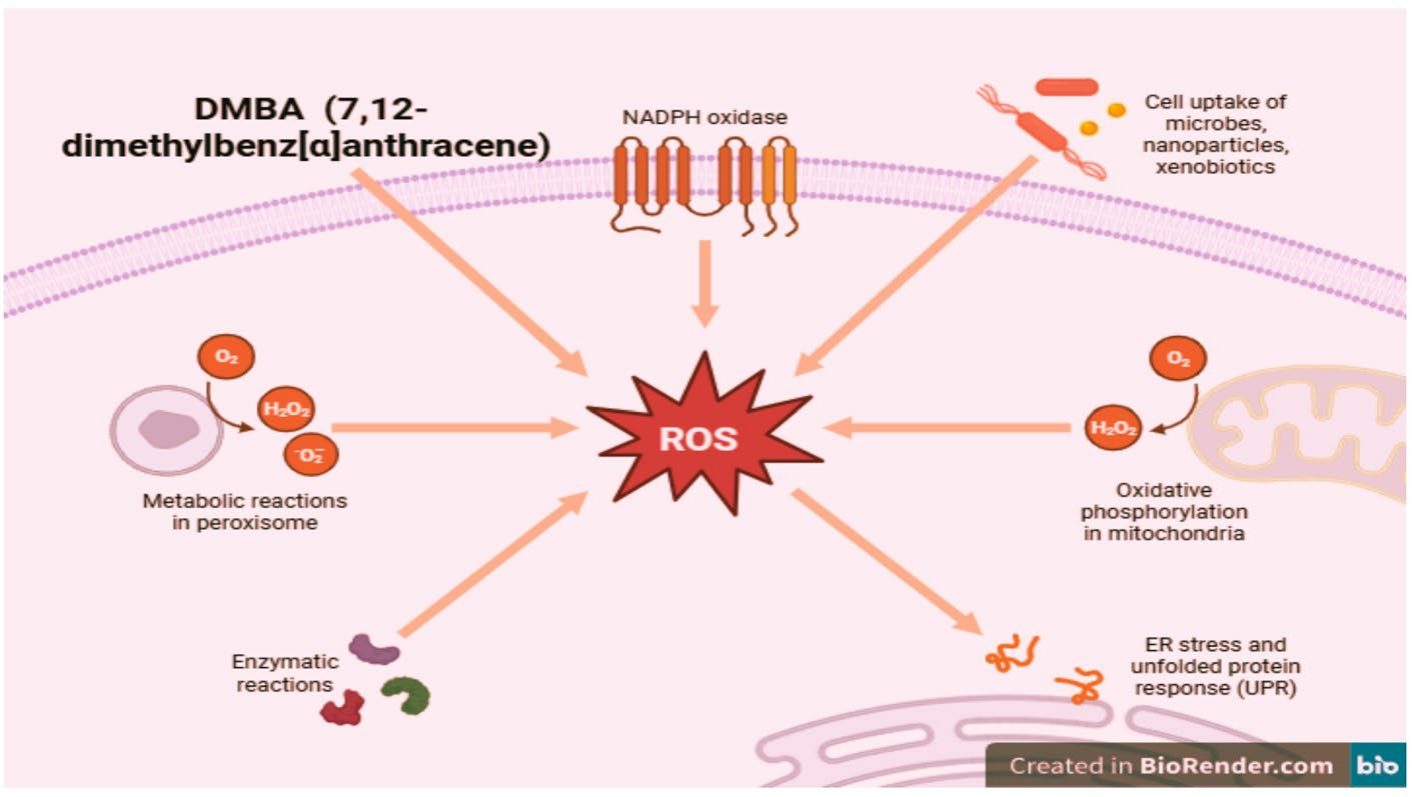
The role of DMBA (7,12-dimethylbenz[α] anthracene) in oxidative stress generation and carcinogenesis of mammary cell

The DMBA model recapitulates many features of human breast carcinogenesis. DMBA undergoes metabolic activation to diol-epoxides that covalently bind DNA, forming bulky adducts and initiating mutagenesis. Beyond direct genotoxicity, DMBA induces oxidative stress (Fig. 9), generates reactive oxygen species (ROS), and activates signaling cascades such as MAPK and NF-κB, collectively driving genomic instability, uncontrolled proliferation, and chronic inflammation [35, 36]. Consequently, this model provides a robust platform for evaluating the chemopreventive and therapeutic efficacy of natural agents such as Carvacrol.

At the intrinsic apoptotic level, Carvacrol significantly modulated mitochondrial regulators of cell death. Our findings demonstrated down regulation of the anti-apoptotic protein BCL2, accompanied by increased expression of p53, p73, and cytochrome c. P53 is a key transcription factor that regulates the expression of genes involved in maintaining genomic stability and preventing abnormal cell proliferation under conditions such as DNA damage, oncogene activation, hypoxia, or loss of cell adhesion [37]– [38]. It exerts its tumor-suppressive function by inducing cell cycle arrest (at G1 and/or G2 phase), senescence, or apoptosis, depending on the type and intensity of stress, cell context, and intracellular environment [39]– [40]. Through apoptosis, p53 eliminates damaged, infected, or excessively proliferating cells, thereby ensuring controlled cell growth in multicellular organisms [41]– [42]. Activated by both intrinsic and extrinsic stress signals, p53 accumulates in the nucleus and promotes either growth arrest or apoptotic cell death, representing a pivotal safeguard against tumor development. Beyond apoptosis, p53 also contributes to DNA repair, differentiation, and angiogenesis regulation, further reinforcing its central role in tumor suppression [43]. The restoration of p53 and p73 further promotes apoptosis by enhancing Bax and suppressing survival signals [44]. These data, together with prior evidence of Carvacrol-induced mitochondrial destabilization and caspase activation in cancer cell models [45], clearly support intrinsic apoptosis activation as a central mechanism of action.

In addition to mitochondrial regulation, Carvacrol engaged extrinsic apoptotic signaling through death receptors. Death receptors, a subgroup of the tumor necrosis factor receptor (TNFR) superfamily, are membrane-bound receptors that transmit apoptotic signals upon ligand binding and play a central role in regulating immune responses, inflammation, and cancer [46]. Eight members have been identified, including TNFR1 (DR1), CD95 (Fas/APO-1/DR2), DR3, TRAILR1 (DR4), TRAILR2 (DR5), DR6, ectodysplasin A receptor (EDAR), and nerve growth factor receptor (NGFR). A common feature of these receptors is the conserved cytoplasmic “death domain” (DD), composed of ~ 80 amino acids, which is essential for apoptotic signaling [47]– [48]. Upon ligand binding, death receptors undergo trimerization and recruit adapter proteins to the DD, initiating signaling cascades that determine cell fate. Two principal complexes are formed: death-inducing signaling complexes (DISCs), assembled on CD95, TRAILR1, or TRAILR2, which recruit and activate caspase-8 to initiate the caspase cascade; and TNFR1-associated complexes, including DR3, DR6, and EDAR, which integrate apoptotic and survival pathways through adapter proteins such as FADD and TRADD [49–52].

In the present study, DMBA exposure impaired death receptor signaling, as reflected by decreased TRAIL and FADD expression with concurrent DR2 elevation. Carvacrol reversed these alterations, restoring TRAIL and FADD expression, suppressing DR2, and markedly elevating TNF-α. Functionally, this reinstated DISC formation and caspase-8 activation, leading to extrinsic apoptosis initiation. Importantly, activated caspase-8 cleaves Bid into tBid, which translocates to mitochondria, triggering cytochrome c release and amplifying mitochondrial apoptosis. Thus, Carvacrol activated extrinsic death pathways while simultaneously reinforcing intrinsic apoptotic signaling, establishing a synergistic dual mechanism [53].

Additionally, TNF-α up-regulation observed with Carvacrol treatment may provide further tumor suppression. While TNF-α is classically a pleiotropic cytokine implicated in inflammation and cancer progression, its elevated expression in this context likely reflects immune-mediated anti-tumor activity and apoptotic signaling. These findings suggest that Carvacrol not only directly induces apoptosis but also potentiates immune-mediated tumor control [54].

This research established that DMBA-induced breast cancer produced substantial increases in liver function enzymes (ALT, AST) as well as creatinine and urea levels, indicating compromised hepatic and renal integrity compared with controls. Among the different regimens, injected Carvacrol produced the most significant improvement, normalizing liver enzyme activities and restoring creatinine and urea values to near-standard limits [55, 56].

Carvacrol demonstrated strong antioxidative and hepatoprotective properties, markedly enhancing enzymatic antioxidants (CAT, SOD, GPx) and non-enzymatic antioxidants (vitamin C, vitamin E, and reduced glutathione) [57]. *Origanum vulgare* L., which contains high concentrations of Carvacrol, has also shown antioxidant and anti-carcinogenic activities, with greater potency against the triple-negative breast cancer cell line MDA-MB-231 compared to the glioblastoma cell line U87 [58, 59].

The carcinogenic activity of DMBA is primarily mediated through oxidative stress and enzymatic production of reactive oxygen species (ROS) and peroxides, which play a critical role in carcinogenesis [60]. In the present study, DMBA exposure elevated oxidative stress, enhanced lipid peroxidation (LPO), and reduced total antioxidant levels relative to normal controls. These findings align with the well-established role of antioxidants as protective agents in carcinogenesis, as they neutralize free radicals and ROS through direct and indirect mechanisms [61]– [62]. Importantly, Carvacrol treatment restored antioxidant capacity and reduced LPO values to near-normal levels, whereas the tumor-bearing group exhibited persistently high LPO. This agrees with previous studies showing that oxidative stress arises when ROS production overwhelms the antioxidant defense system [63].

ROS and free radicals are known to damage cellular proteins, lipids, and nucleic acids, thereby accelerating tumor initiation and progression. Plant-derived essential oils, including Carvacrol, function as natural antioxidants that scavenge free radicals and protect against genetic mutations, tumorigenesis, and aging [64]– [65]. Carvacrol has also been reported to alleviate oxidative stress in the brain, liver, and kidneys of rats [66]. Its administration elevates the activity of antioxidant enzymes—superoxide dismutase, catalase, glutathione peroxidase, and glutathione reductase—while increasing glutathione levels. At the same time, it lowers lipid peroxides and serum markers of tissue injury (AST, ALT, ALP, LDH, and γ-GT) [67]. Collectively, these antioxidant, hepatoprotective, and DNA-protective properties of Carvacrol [68] contribute to its observed anticancer efficacy in DMBA-induced breast carcinogenesis.Taken together, our results demonstrate that Carvacrol exerts a multi-targeted anticancer effect by activating both intrinsic (p53/p73, BCL2 down-regulation, cytochrome c release) and extrinsic (TRAIL, FADD, DR2, TNF-α, caspase-8) apoptotic pathways, while simultaneously mitigating oxidative stress and restoring liver and kidney biomarkers. This integrated mechanism culminates in robust tumor suppression, with injection delivery yielding the strongest outcomes.

Importantly, the current findings add novel insights beyond previous reports. While earlier studies have described the antioxidant and pro-apoptotic activities of Carvacrol in the current study, is the first to demonstrate that injection delivery markedly outperforms oral or mixed administration, highlighting the critical role of bioavailability in therapeutic efficacy. Moreover, the comprehensive modulation of immune-related death receptor markers (DR2, TRAIL, FADD), in parallel with intrinsic apoptotic regulators, underscores a unique dual engagement of apoptosis that has not been fully reported before. These distinctions clearly set our results apart from prior research and establish Carvacrol injection as a promising and superior therapeutic approach in DMBA-induced breast carcinogenesis.

## Conclusion

The present study demonstrates that Carvacrol demonstrates a multi-faceted therapeutic potential through combined regulation of apoptosis, oxidative stress, and organ function integrity. These findings support Carvacrol as a promising natural therapeutic candidate against breast carcinogenesis and warrant further exploration in translational and clinical studies.

### Future directions and limitations

Future plans include the search and development of reliable breast models, whether in vitro, in vivo, or in silico; applying robust methodology to define a standard and safe dose, and clarify its exact mechanisms of action. Immunohistochemistry is one very powerful tool needed to understand the mechanism of Carvacrol action in primary breast cancer and its anti-proliferative effect on breast cancer metastasis as well as to support the ELISA-based quantification thus offering more validation of the results. We also look forward to investigate the role of Nanotechnology as an innovative strategy to improve the efficacy and targeted delivery of Carvacrol therapeutics in breast cancer. These investigations work to enhance the translational relevance of Carvacrol in breast cancer therapy.

## Data Availability

All data generated or analyzed during this study are included in this published article.

## Abbreviations

ALT: Alanine aminotransferases
ANOVA: Analysis of variance
AST: Aspartate aminotransferases
BC: Breast cancer
Bcl-2: reduces B-cell leukemia/lymphoma 2
CV: Carvacrol
DMBA: 7,12-Dimethylbenz(a)anthracene
DNA: Deoxyribonucleic acid
DR2: Dopamine receptor 2
FADD: Fas-associated death domain
GPx: Glutathione peroxidase
GSH: Reduced glutathione
H&E: Hematoxylin and Eosin
LPO/MDA: Malonaldialdehyde
MMP: Mitochondrial membrane potential
P53& P73: tumor protein genes
PARP: Poly-ADP-ribose polymerase
ROS: Reactive oxygen species
SOD: Superoxide Dismutase
SPSS: Statistical Package for the Social Sciences Software
TNF-α: tumor necrosis factor alpha
TRAIL: tumor necrosis factor apoptosis inducing ligand
WHO: The World Health Organization
